# Methyl Jasmonate Effect on Betulinic Acid Content and Biological Properties of Extract from *Senna obtusifolia* Transgenic Hairy Roots

**DOI:** 10.3390/molecules26206208

**Published:** 2021-10-14

**Authors:** Tomasz Kowalczyk, Przemysław Sitarek, Anna Merecz-Sadowska, Monika Szyposzyńska, Aleksandra Spławska, Leslaw Gorniak, Michał Bijak, Tomasz Śliwiński

**Affiliations:** 1 Department of Molecular Biotechnology and Genetics, University of Lodz, Banacha 12/16, 90-237 Lodz, Poland; 2 Department of Biology and Pharmaceutical Botany, Medical University of Lodz, Muszynskiego 1, 90-151 Lodz, Poland; przemyslaw.sitarek@umed.lodz.pl; 3 Department of Computer Science in Economics, University of Lodz, 90-214 Lodz, Poland; anna.merecz-sadowska@uni.lodz.pl; 4 CBRN Reconnaissance and Decontamination Department, Military Institute of Chemistry and Radiometry, Antoniego Chrusciela “Montera” 105, 00-910 Warsaw, Poland; m.szyposzynska@wichir.waw.pl (M.S.); a.splawska@wichir.waw.pl (A.S.); 5 Biohazard Prevention Centre, Faculty of Biology and Environmental Protection, University of Lodz, Pomorska 141/143, 90-236 Lodz, Poland; leslaw.gorniak@biol.uni.lodz.pl (L.G.); michal.bijak@biol.uni.lodz.pl (M.B.); 6 Department of Medical Biochemistry, Medical University of Lodz, Mazowiecka 6/8, 92-215 Lodz, Poland; tomasz.sliwinski@biol.uni.lodz.pl

**Keywords:** *Senna obtusifolia*, transgenic hairy roots, sprinkle bioreactor, methyl jasmonate, cytotoxicity, cancer cells

## Abstract

It is known that *Senna obtusifolia* has been used in medicine since ancient times due to the content of many valuable compounds with a pro-health effect. One of them is betulinic acid, which is a pentacyclic triterpene with antimalarial, antiviral, anti-inflammatory and anticancer properties. In this work, a continuation of our previous research, an attempt was made to increase the level of betulinic acid accumulation by the cultivation of transgenic hairy roots that overexpress the squalene synthase gene in a 10 L sprinkle bioreactor with methyl jasmonate elicitation. We present that the applied strategy allowed us to increase the content of betulinic acid in hairy root cultures to the level of 48 mg/g dry weight. The obtained plant extracts showed a stronger cytotoxic effect on the U87MG glioblastoma cell line than the roots grown without elicitors. Additionally, the induction of apoptosis, reduction of mitochondrial membrane potential, chromosomal DNA fragmentation and activation of caspase cascades are demonstrated. Moreover, the tested extract showed inhibition of topoisomerase I activity.

## 1. Introduction

Plant in vitro cultures are a rich source of biologically active compounds of natural origin, gaining more and more importance in many areas of human life [1,2,3,4,5,6]. The promising results of numerous studies in this area allow such findings to be widely used as the basis for or supplement to the therapy of many serious diseases. Their ability to produce secondary metabolites is also used in many industries as the basis for various technologies [7,8,9]. The wide possibility of the efficient cultivation of plant cells, tissues or organs in the in vitro systems means that strategies based on plant cells are now gaining popularity. Well-developed methods of culturing plant material under strictly controlled conditions enable intensive growth and, consequently, further optimization of their productivity. One of the methods to increase such productivity is elicitation, i.e., exogenous administration of factors that cause stress to plant cells, thus increasing the production of selected secondary metabolites. The mechanism of action of elicitors is generally based on the recognition of signals by plant cell membrane receptors and, consequently, on the stimulation of defense reactions in the form of synthesis and accumulation of the appropriate compound. So far, many examples have been reported in which the use of such an approach resulted in higher productivity [10,11,12]. Akhgari et al. [13], studying the effects of different doses of methyl jasmonate (MeJA) on the accumulation of terpenoid indole alkaloids in the hairy roots of the *Rhazya stricta,* showed an increased content of alkaloids compared to non-elicited cultures. Additionally, Wei et al. [14] showed that kaurene synthase-like gene overexpression combined with methyl jasmonate treatment enhanced tanshinone production from *Salvia miltiorrhiza* hairy roots. The combination of the currently available strategies for genetic engineering and transformation of plants, together with the use of optimal growth conditions and elicitation, often bring very good results related to the high productivity of plant in vitro systems. Optimal conditions for the cultivation of hairy roots on a larger scale can often be ensured by growing them in specially designed bioreactors that allow strict conditions to be controlled and the entire culture cycle to be automated. Bioreactors, as culture systems that provide a sterile environment for growing cells, tissues and plant organs, are used to optimize and monitor the conditions of the culture environment [15]. Hairy roots are specific structures of complex nature, which have many limitations when grown on a large scale. A big challenge in their propagation is a limited access to oxygen or nutrients from the culture medium. Ensuring optimal aeration, temperature and pH often contributes to the intensive growth of the cultivated material and, consequently, to obtaining the desired amount of biologically active compounds. The combination of many strategies leading to an intensive growth in biomass and the biosynthesis of valuable secondary metabolites usually provides satisfactory results. Hao et al. [16] showed that methyl jasmonate (MeJa), in combination with salicylic acid (SA), can significantly affect the accumulation of tanshinone and the expression of biosynthetic genes in hairy roots, overexpressing the geranylgeranyl diphosphate synthase overexpression line (*SmGGPPS*) in *Salvia miltiorrhiza.* The results of our previous work have shown that a combination of many strategies, such as the genetic manipulation and cultivation of hairy roots in a bioreactor, can lead to a significant increase in valuable secondary metabolites in *Senna obtusifolia* hairy root cultures [17]. This valuable plant is widely used in traditional Chinese medicine to prevent and treat diabetes, hyperlipidemia, hypertension, photophobia, headache, dizziness, liver and Alzheimer’s disease [18,19]. Terpenoid, the betulinic acid found in this plant, has even been tested as an anti-HIV drug [20]. An extract of transgenic *Senna obtusifolia* L. hairy roots with overexpression of the *Pgss1* gene, in combination with a chemotherapeutic agent, induces apoptosis in the leukemia cell line by activating apoptotic-related protein expression. Additionally, an extract from these roots grown in the bioreactor has been shown to have a cytotoxic and antiproliferative effect on U87MG glioblastoma cells and to alter the level of selected apoptotic proteins. Antimicrobial and moderate antiviral properties have also been demonstrated for this extract [17].

In this work, we present results that are a continuation of our previous research on an attempt to obtain transgenic *Senna obtusifolia* hairy roots with overexpression of the gene encoding squalene synthase, grown in a sprinkle bioreactor with elicitation, in order to increase the content of betulinic acid. Our results allowed us to confirm the higher content of this valuable metabolite in the tested extracts and stronger biological properties than those of the transformed roots induced by *Rhizobium rhizogenes*.

## 2. Results

### 2.1. Transgenic Hairy Root Growth Index

**Figure 1:**
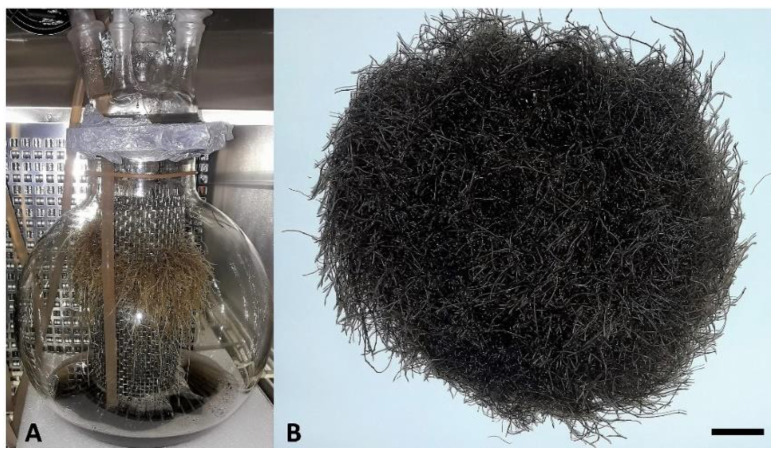
(**A**) Transgenic hairy roots cultivated in 10 L sprinkle bioreactor after 25 days of culture (before elicitor application). (**B**) Hairy roots after 35 days of culture in bioreactor and 10 days elicitor treatment; bar = 1 cm.

The growth index of the transgenic hairy roots grown in the bioreactor (Figure 1A) was 22.1 after 35 days of cultivation, which was lower than that obtained earlier (247 g) without the use of elicitation. In this work, the optimal cultivation conditions turned out to be those with 45 s of medium application and 90 s of break between each application (data not shown). The use of a sprinkle nozzle situated above the growing roots also provided better results due to better penetration of the culture medium into the dense biomass of growing hairy roots. The hairy roots, after 10 days of cultivation with 100 µM MeJA, showed a much darker color than before the addition of the elicitor, while remaining alive (Figure 1B).

### 2.2. Effect of Elicitation on Protein Profile of Senna Obtusifolia Transgenic Hairy Roots

**Figure 2:**
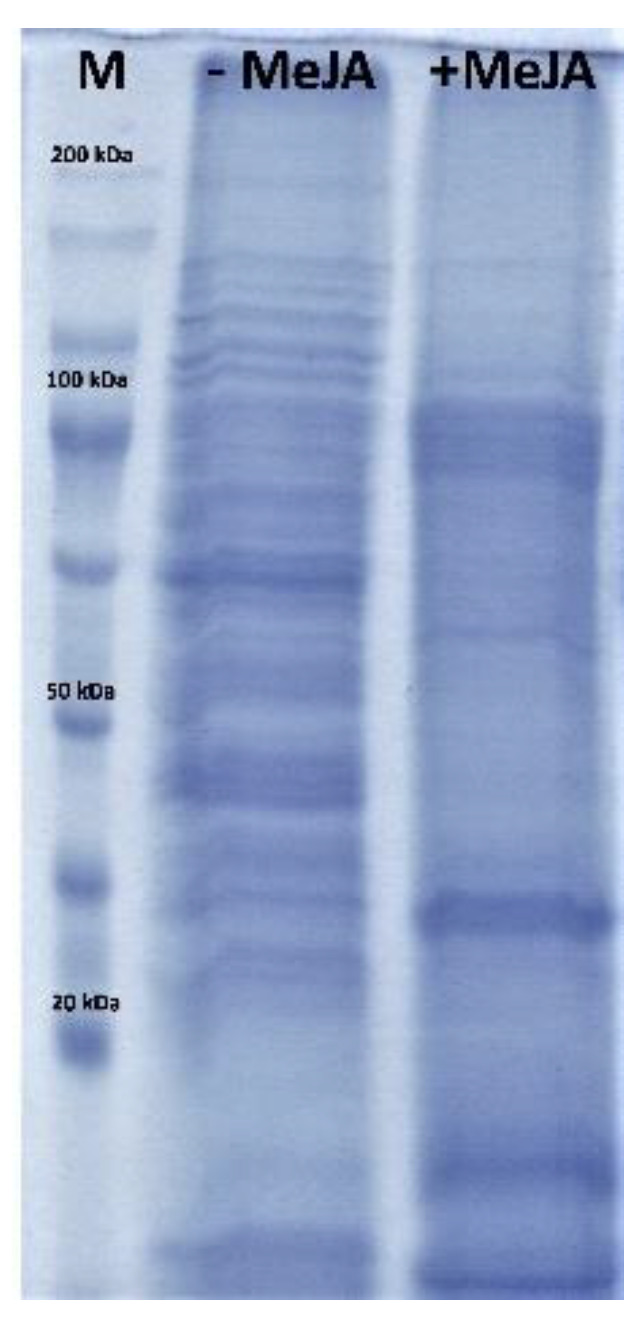
SDS-PAGE analysis of protein extracts from transgenic hairy roots of *Senna obtusifolia*. separated by 10% SDS-PAGE. M, standard marker proteins; −MeJA and +MeJA, protein samples isolated after 35 days culture without and with methyl jasmonate, respectively.

In this work, the influence of the applied elicitor (MeJA) on the protein profile of the transgenic hairy roots grown in the bioreactor is preliminarily investigated. The analyzed protein extracts derived from the transgenic hairy roots with the use of 100 µM MeJA and without the elicitor showed differences in the protein profile. A qualitative difference in the extracted protein pattern between untreated and MeJA-treated hairy roots was observed on SDS-PAGE. There was a significant increase in proteins with a mass of about 100 kDa and 30 kDa, respectively (Figure 2).

### 2.3. HPLC Analysis

The HPLC analysis showed that the analyzed extract from the transgenic hairy roots grown in a 10 L bioreactor with the use of MeJA contained 48 mg/g dry weight (DW) of betulinic acid. These results show an increase in the content of this metabolite, compared to transgenic hairy roots grown in a bioreactor without the use of an elicitor. In our study, we also cultivated transgenic (after *R. rhizogenes* transformation, without genetic construct) hairy roots under identical conditions without the use of MeJA. The content of betulinic acid in these roots was significantly lower (data not shown). Therefore, we chose only the extract from transgenic hairy roots grown with MeJA for further biological research (Figure S1).

### 2.4. Effect of Transgenic Hairy Roots of Senna obtusifolia Extracts on U87MG, DU-145 and A549 Cancer Cells Lines

**Figure 3:**
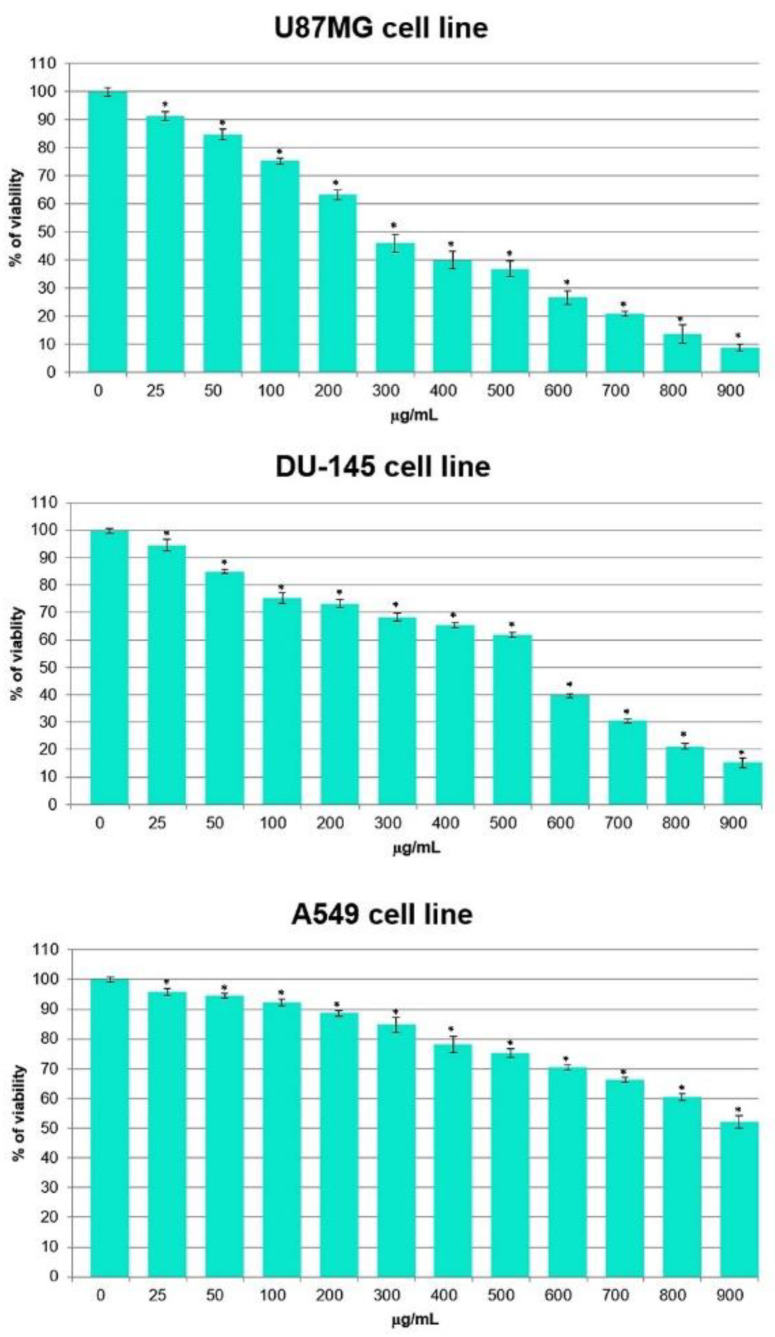
Cytotoxic effect of *S. obtusifolia* transgenic hairy root extract after MeJA treatment on the viability of three cancer cell lines. Data for each cell lines are presented at concentrations of 0–900 µg/mL after incubation for 24 h. All experiments were performed in triplicate and results are expressed as mean ± SD. * *p* < 0.05 transgenic hairy root extract vs. untreated cells.

The result of the MTT revealed that the tested transgenic hairy root extract decreased the percentage of viability for all of the cells, but to a different extent and depending on the tested cell line (Figure 3). The extract was found to induce more cytotoxicity towards cancer cell lines U87MG, DU-145 and A549 with IC_50_ = 250 µg/mL, 550 µg/mL and 920 µg/mL, respectively. The U87MG line was the most sensitive to the effect of the tested extract; therefore, this line was chosen for further studies. In turn, for the hairy root extract in the tested concentration range, IC_50_ was not reached for the A549 cancer cell line.

### 2.5. Apoptosis and Necrosis Determination Using Flow Cytometry

**Figure 4:**
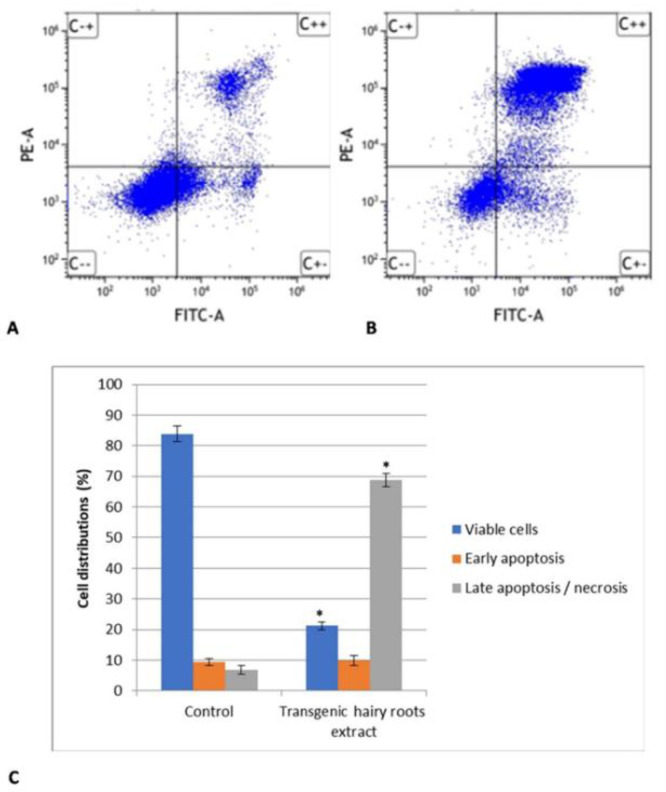
Apoptosis/necrosis induction by transgenic *S. obtusifolia* hairy root extract at the IC_50_ concentration on U87MG cell line for 24 h determined by Annexin V–FITC/PI flow cytometry. Graphs represent distribution of the cells in viable state, early and late apoptosis and necrosis in (**A**) control, untreated U87MG cells and (**B**) 24 h after treatment with MeJA extract. (**C**) Diagram shows percentage of cell populations divided into viable cells, early apoptotic, late apoptotic/necrotic cells. Data represent the mean ± SD (*n* = 3). * *p* < 0.05 transgenic hairy root extract vs. control.

The results of the apoptosis analysis are presented in Figure 4. The transgenic hairy root extract *of S. obtusifolia* in IC_50_ concentration after application of MeJA significantly reduced the number of viable cells while increasing the number of apoptotic cells, compared to untreated cells. The hairy root extract had an effect with 68% late apoptotic/necrotic cells for the U87MG cell line.

### 2.6. Loss of Mitochondrial Membrane Potential (MMP)

**Figure 5:**
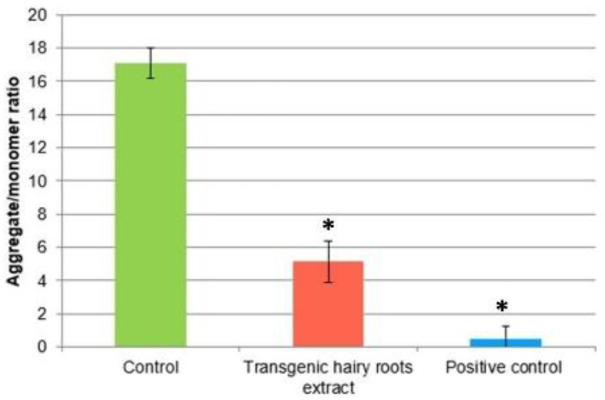
Changes in mitochondrial membrane potential (MMP) of U87MG cells incubated with transgenic hairy root extract of *S. obtusifolia* after MeJA for 24 h. MMP is expressed as ratio of 530 nm/590 nm to 485 nm/538 nm (aggregates to monomer) fluorescence as quantified with a fluorescent plate reader after JC-1 staining. Results are presented as means ± SD. * *p* < 0.05 transgenic hairy root extract vs. control.

The alteration of mitochondrial potential is one of the next events in the apoptotic pathway. This study shows the effect of *S. obtusifolia* extract (IC_50_ concentration) tested on this mitochondrial dysfunction in U87MG cells after 24 h of treatment. Similarly, 10 μM m-chlorophenylhydrazone carbonyl cyanide (CCCP) was used as a positive control. The MMP analysis results are presented in Figure 5. Our results revealed that the tested extract significantly reduced the MMP in tested cells. This indicates that apoptosis induction involves the mitochondrial pathway, suggesting depolarization and ΔΨm collapse by tested hairy root extract after MeJA treatment.

### 2.7. Chromosomal DNA Fragmentation

**Figure 6:**
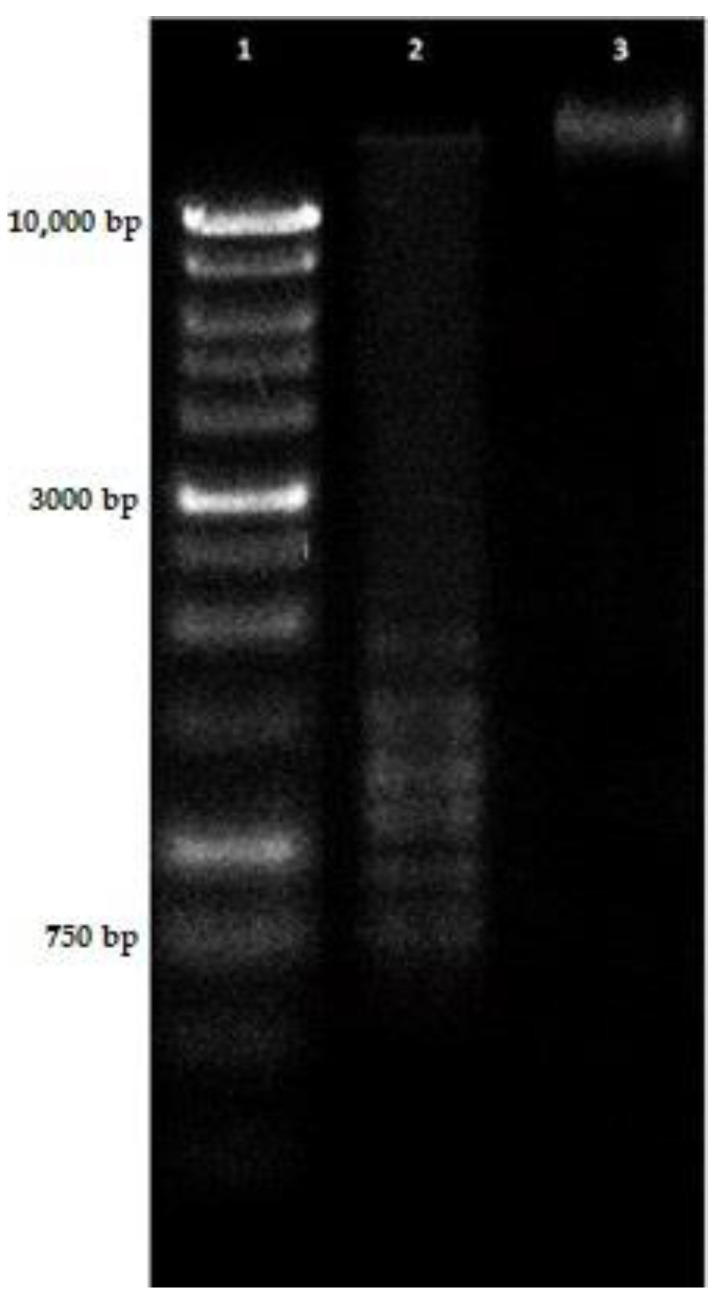
Electrophoresis of DNA extracted from U87MG cells, after treatment with transgenic hairy root extract after MeJA at IC_50_ concentration after 24 h treatment. **Lane 1,** DNA molecular weight marker; **Lane 2**, DNA isolated from cells treated with transgenic hairy root extract for 24 h; **Lane 3**, DNA isolated from cells treated with 0.1% DMSO for 24 h.

One of the hallmarks of apoptosis is DNA fragmentation. In our studies, U87MG cells treated with transgenic hairy root extract after MeJA at IC_50_ concentration induced a smear of DNA fragmentation with smaller DNA fragments, compared with the used DNA ladder that extends from 10 kbp to 250 bp. The control for U87MG cells, treated with 0.1% Dimethyl sulfoxide, showed no fragmented DNA and the smear pattern of damaged DNA (Figure 6).

### 2.8. DNA Damage Measured by Comet Assay

**Figure 7:**
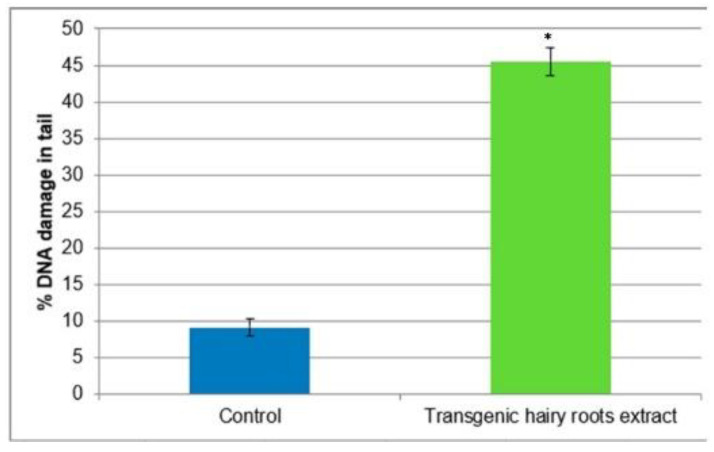
Analysis of DNA damage as measured by comet assay in U87MG cells treated with transgenic hairy root extracts of *S. obtusifolia* after 24 h. Each value represents mean ± SD of three separate experiments. * *p* < 0.05 transgenic hairy root extract vs. control.

The comet assay was used to test the ability of transgenic hairy root extract of *S. obtusifolia* after MeJA elicitation to induce DNA double-strand breaks after 24 h in the U87MG cell line. Our studies revealed that the tested extract induced DNA damage in U87MG cells. The mean percentage of DNA in the tail was 45% (Figure 7).

### 2.9. Influence on the Caspase-3/7 Activity

**Figure 8:**
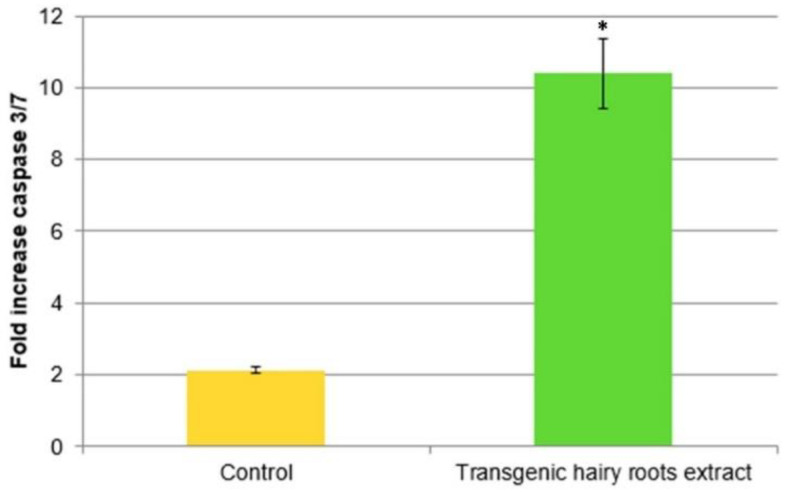
Caspase-3/7 enzymatic activity of U87MG cells and fold increase in activity after 24 h treatment of with IC_50_ of the transgenic hairy root extract of *S. obtusifolia* after MeJA. All data expressed as mean ± standard deviation (SD) at a significance level of * *p* < 0.05.

To investigate whether the apoptosis effect was induced by the transgenic hairy root extract of *S. obtusifolia* after treatment with MeJA, the caspase-3/7 activity was examined in U87MG cells treated at the concentration of IC_50_ values of the extract for 24 h (Figure 8). The tested extract showed a significant effect on activation of caspase-3/7, up to 10.5-fold that of untreated cells.

### 2.10. Topoisomerase I Inhibition Assay with Hairy Root Extract

**Figure 9:**
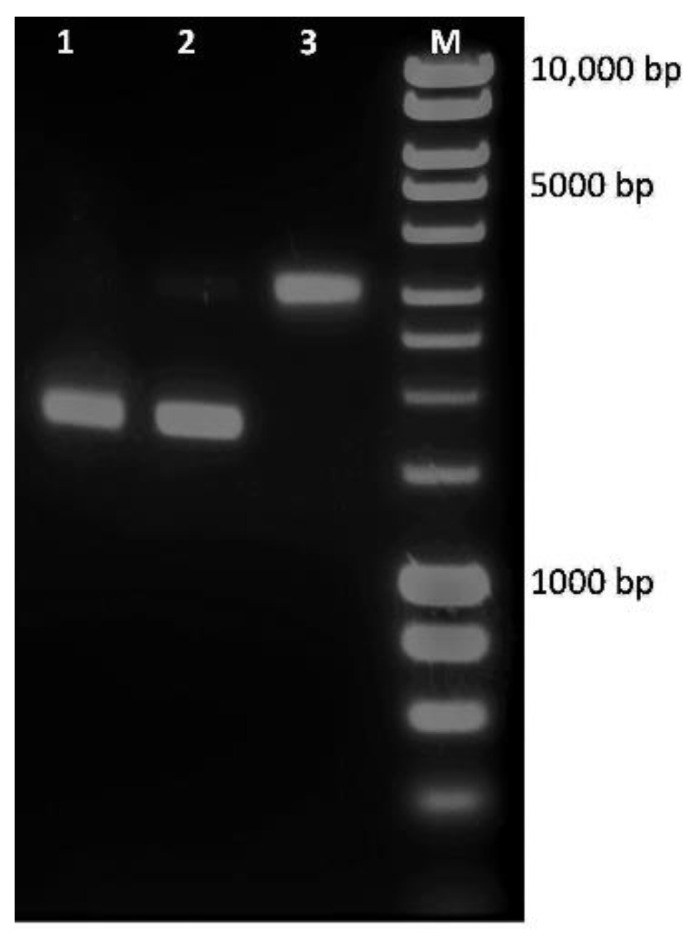
The effect of transgenic hairy root extract of *S. obtusifolia* on the relaxation of pUC19 supercoiled DNA by topoisomerase I. Lane **1**, supercoiled plasmid DNA, Lane **2,** supercoiled plasmid DNA treated with topoisomerase I and hairy root extract after MeJA application Lane **3**, plasmid DNA treated with topoisomerase I; Lane **M**, DNA molecular weight marker.

The topoisomerase I inhibition assay after treatment of transgenic hairy root extract after application of MeJA was performed to evaluate the ability to inhibit topoisomerase I activity. Figure 9 indicates that this extract was able to inhibit pUC19 plasmid DNA relaxation related to topoisomerase I activity inhibition.

## 3. Discussion

Green biotechnology deals with the development and use of environmentally friendly solutions and is often an alternative to traditional processes, e.g., agriculture or horticulture [21,22]. An example is the design of transgenic plants that are genetically modified to improve their flavor and increase their resistance to pests and diseases, or enhance the production of many valuable compounds by a technological application that uses biological systems or their derivatives to produce or modify products or processes for a specific application [23,24,25]. One of the leading trends in modern plant biotechnology is the attempt to increase the production of valuable secondary metabolites in response to the growing demand. Hairy roots are an interesting and efficient model that allows for many modifications and ensures a rapid increase in biomass [26,27,28]. Additionally, the possibility of growing them under strictly controlled conditions makes them a great alternative to the constantly diminishing natural resources that are the source of valuable, biologically active compounds used in pharmacology.

Another commonly used strategy, that brings the expected results and allows the productivity of plant cultures in vitro to be additionally increased, is the use of a wide range of biotic and abiotic elicitors [29,30,31,32]. Additionally, by manipulating metabolic pathways (introducing new genes or silencing selected ones), it is possible to create efficient plant-based production systems. Plant cells or tissue cultures obtained in this way can be transferred to bioreactors, allowing them to be cultivated on a larger scale, which can directly lead to a further increase in productivity and even their use on an industrial scale [33,34]. The entire set of factors described above makes it possible to develop highly efficient plant culture systems, the metabolites of which can be used in the pharmaceutical and medical industries [35,36].

The aim of our study is to present, for the first time, the influence of MeJA on the production of betulinic acid in *S. obtusifolia* transgenic hairy root cultures with overexpression of the *PgSS1* gene grown in a bioreactor and to evaluate the biological properties of the obtained extract.

The first step of our research project was an attempt, for the first time, to cultivate *S. obtusifolia* transgenic hairy roots in a 10 L sprinkle bioreactor and to use methyl jasmonate as an elicitor to increase the level of betulinic acid production. Methyl jasmonate (MeJA) is a well-known and widely used elicitor that causes an increased production of many valuable secondary metabolites. It is naturally produced in plants, especially as the ’stress hormone’ in response to an insect attack [37,38]. Our results confirmed the possibility of increasing the level of the tested metabolite in relation to the previously applied system, which found betulinic acid at a concentration of 4.21 mg/g and 38.12 mg/g dry weight for transformed (without genetic construct) and transgenic (overexpressing the squalene synthase gene) hairy roots, respectively. In this study, we present the possibility of enhancing the content of this metabolite after additional elicitation with methyl jasmonate at the concentration of 48.8 mg/g DW, which is an increase in production by 28% in relation to our previous results. At the same time, a decrease in the growth index (GI) of roots with the use of elicitor was observed compared to the roots grown without MeJA. The possibility of increasing the content of valuable phytochemicals such as triterpenes is presented by Mangas et al. [39], who demonstrated that methyl jasmonate may be used as an inducer of enzymes involved in the triterpenoid synthesis in plant in vitro cultures. Hajati et al. [40] showed that the use of biotic and abiotic elicitors, including MeJA, may lead to an improved accumulation of betulin and betulinic acid in the cell culture of *B. pendula*. The use of MeJA allowed the level of betulinic acid to increase two-fold in the tested suspension cultures, compared to the control. Other authors also showed a two-fold increase in betulinic acid in callus cultures of different *Ocimum* species after MeJA treatment [41]. On the other hand, Park et al. [42] showed an increase in the content of betulinic acid in *Morus alba* hairy roots after the use of yeast extract with a similar yield to MeJA compared to the control. The above studies show that the use of methyl jasmonate in plant cultures may cause the growth of pentacyclic triterpenes, including betulinic acid, which is consistent with our results. The novelty of this work consists in the use of an elicitor for the first time for *S. obtusifolia* transgenic hairy roots grown in a bioreactor. The application of metabolic engineering and large-scale cultivation in a bioreactor, using elicitation, may lead to the development of a more efficient system for the production of secondary metabolites in in vitro plant cultures. In line with our previous studies, in which we showed a higher content of betulinic acid in transgenic hairy roots with overexpression of squalene synthase, growing plant transgenic cultures on a larger scale allows us to increase their productivity. Additionally, our results showed a decrease in biomass of transgenic hairy roots grown in the bioreactor after elicitation, compared to untreated hairy roots. These results are consistent with Wielanek et al. [43], who showed a decrease in the biomass of *Tropaeolum majus* L. hairy roots after the application of MeJA. Kang et al. showed a decrease in the biomass of *Scopolia parviflora* hairy roots after treatment with methyl jasmonate [44]. Observations on the decrease in biomass after treatment with the MeJA elicitor may be related to the inhibition of the primary metabolism and activation of the secondary metabolism. This may result in an inverse relationship between biomass production and accumulation of the secondary metabolite and, consequently, inhibition of hairy root growth. According to our results, there was an increase in betulinic acid content and a reduction in the biomass of hairy roots by 10.5%. Additionally, the analyzed protein extracts, derived from transgenic hairy roots with the use of 100 µM MeJA and without the elicitor, showed qualitative differences in the protein profiles. Further analyses are necessary to identify individual proteins and their possible function in transgenic hairy roots in response to the elicitor and selected secondary metabolites’ biosynthesis.

The next step of our research project was to evaluate the cytotoxicity of the tested extracts derived from transgenic hairy roots of *S. obtusifolia* with overexpression of squalene synthase after MeJA treatment grown in a 10 L sprinkle bioreactor on three cancer cell lines (glioblastoma multiforme U87MG, human lung adenocarcinoma A549 and human prostate carcinoma DU-145). One of the above cell lines (U87MG) was tested in our previous work, where the tested extract showed a cytotoxic effect at IC_50_ = 360 µg/mL. In this study, the IC_50_ for the same cell line was 250 µg/mL. We suggest that a stronger cytotoxic effect may be associated with the use of the MeJA elicitor for the cultivation of hairy roots in a bioreactor, which may increase the content of betulinic acid—responsible for this effect alone or in synergy with the compounds present in the extract. Our previous work also showed the effect of the tested extract on the induction of apoptosis in U87MG cells. In addition, a cytotoxic effect was also demonstrated for the DU-145 cell line, whereas the weakest influence of the tested extract was demonstrated for the A549 cell line. Based on the obtained results, we suppose that differences in the cytotoxic effect of the extracts from hairy roots may result from the different origins of the neoplastic cell lines, determining their different sensitivity to the phytochemicals contained in the studied extract. This is the first study showing the cytotoxic effect of the extract of *S. obtusifolia* transgenic hairy roots grown in a bioreactor using the U87MG tumor cell line and the application of an elicitor. Yaozu et al. [45] demonstrated the cytotoxic effect of betulinic acid on the same tumor cells depending on the dose. We suggest that the observed effect may be related to the higher content of betulinic acid in the tested extract. Continuing the previous research, confirming the induction of apoptosis by changing the level of apoptosis-related proteins in U87MG cells after the transgenic hairy root extract, in this work, we extended the methodology to include additional studies, thus confirming the observed phenomenon through the genotoxic effect, change of mitochondrial membrane potential, caspase cascade activation and DNA fragmentation.

Caspase-3 and caspase-7 are activated during apoptosis, independent of the factor initiating cell death. Our research experiments showed a significant activation of caspases after treatment with the tested extract of transgenic hairy roots. Research papers showed that the activation of the caspase cascade (group of cysteine proteases responsible for the execution of cellular death) is one of the key indicators of apoptosis in cancer cells [46,47]. Our results (DNA fragmentation, activation of the caspase cascade and change in the mitochondrial membrane potential) suggest that the tested extract may activate apoptosis on the intrinsic and/or extrinsic pathway. We confirmed the induction of apoptosis in the tested cells by increasing the level of DNA damage. The disruption of mitochondrial membrane potential (ΔΨm) is one of the earliest intracellular events that occur following the onset of apoptosis [48]. As of yet, there are no data in the literature about the influence of the extract derived from transgenic hairy roots of *S. obtusifolia* after MeJA treatment on cancer cells’ DNA fragmentation. Based on the publication by Goswami et al. [49], we suggest that the higher concentration of betulinic acid detected in the extract in our study may induce DNA damage of U87MG cells. Additionally, other studies confirm that the transgenic hairy root extract can damage the DNA of U87MG cancer cells [50].

The last stage of our research was to test the ability to inhibit topiosomerase I in the tested samples with the use of plasmid DNA. Plant compounds (alkaloids, terpenoids, polyphenols or quinones) are known to inhibit the activity of DNA topoisomerase, which is the clinical target of many potential anticancer drugs [51,52,53,54]. Currently, the routinely used part of anti-neoplastic drugs is based on the inhibition of the activity of topoisomerases. However, they exhibit relatively high toxicity. Therefore, there is a great need to develop alternative solutions using compounds of natural origin, especially plant-derived. Our preliminary analyses showed that the extracts derived from the transgenic hairy roots of *S. obtusifolia* overexpressing the squalene synthase gene grown in a bioreactor using MeJA elicitation have the ability to inhibit topoisomerase I activity. The currently observed intensification in research related to the development of new and alternative systems for the production of valuable plant secondary metabolites is related to the growing global demand. The dramatically increasing number of cancer cases forces us to obtain such compounds from plant cultures in vitro. Fortunately, extensive knowledge and technical capabilities allow researchers to develop efficient systems of plant in vitro cultures more and more often. In this work, we present a combination of genetic manipulation, elicitation and large-scale cultivation of hairy roots, resulting in the development of a fairly efficient plant system.

## 4. Materials and Methods

### 4.1. Establishment of Hairy Root Cultures

The in vitro cultivation of *Senna obtusifolia* and the induction of hairy roots with *Rhizobium rhizogenes* A4 was presented earlier [55]. Briefly, 14-day-old seedlings grown in vitro on MS medium were inoculated with a suspension of *Rhizobium rhizogenes* A4 and incubated for 3 days. After this time, the plant material was washed with an antibiotic solution and the first hairy roots appeared 10 days after transformation. Hairy root culture was carried out in the dark at 25 °C.

### 4.2. Optimization of Hairy Root Culture Conditions in the Bioreactor

In order to further optimize the hairy root growth conditions in the 10 L sprinkle bioreactor, different periods of sprinkling the growing roots with the nutrient solution and different intervals between sprinkling were used. For this purpose, 15, 30 and 45 s sprinkling of roots and 30, 90 and 180 s breaks were tested. In addition, the effectiveness of sprinkling the roots from the top and bottom was checked using a special nozzle situated above the roots and under the stainless-steel growth basket.

### 4.3. Elicitor Treatment

Hairy roots were grown in the sprinkle bioreactor as previously described [17]. In this study, we attempted to increase the content of betulinic acid by applying MeJA on a hairy root culture. Hairy roots were grown in the bioreactor for 25 days. After this time, methyl jasmonate was applied to the culture at a concentration of 100 µM. The culture with the elicitor was carried out for 10 days under the same conditions. Then, the hairy roots were harvested. All treatments were performed in triplicate.

### 4.4. Hairy Roots Growth Index Determination

A growth index (GI) of hairy roots growing in bioreactor was established as

(1) $$GI=\frac{\mathrm{fresh}\;\mathrm{weight}\;\mathrm{after}\;35\;\mathrm{days}\;\mathrm{of}\;\mathrm{culture}}{\mathrm{fresh}\;\mathrm{weight}\;\mathrm{of}\;\mathrm{the}\;\mathrm{inoculum}}$$

### 4.5. Hairy Root Protein Isolation

Hairy roots from the last day of culture were used for protein isolation. For this, 0.5 g of biomass was homogenized in liquid nitrogen using a mortar and pestle and then 1.5 mL of an appropriate extraction buffer (1.5% sucrose, 5% 2-mercaptoethanol, 2.5% Trizma base and 2% SDS, pH = 8.5) was added and homogenized for 5 min. The homogenates were then collected into Eppendorf tubes and centrifuged at 6000× *g* for 30 min at 4 °C. The supernatants were collected in new tubes and used for further analyses. The protein concentration was determined by the Bradford method in order to use equal protein concentrations for the analyses [56].

### 4.6. SDS-PAGE Protein Electrophoresis

Separation of the isolated proteins was carried out in a polyacrylamide gel according to the Laemmli system [57]. The resolving gel was 10% and the stacking gel was 5%. The Bio-Rad Mini Protean 3 Cell gel electrophoresis system was used for separation. A total of 20 µg of protein per well was used for separation. Protein samples were mixed with a loading buffer (250 mM Tris–HCl, pH 6.8, 8% SDS, 30% glycerol, 5% β-mercaptoethanol and 0.04% bromophenol blue) and heated at 95 °C for 10 min before separation. Electrophoresis was performed at room temperature for approximately 1 h using constant voltage (200 V) in 1X Tris-glycine-SDS buffer (TG-SDS) until the dye front reached the end of the gel. Subsequently, the gel was stained using Colloidal Coomassie G-250 Staining, according to Dyballa and Metzger [58].

### 4.7. Plant Extract Preparation

Preparation of plant extracts for analyses was carried out according to the protocol presented previously [17]. Briefly, the extracts of transgenic hairy roots grown in a sprinkle bioreactor with MeJA were prepared as follows: A total of 20 g of dry root mass was extracted for 15 min with 80% (*v*/*v*) aqueous methanol (1000 mL) at 35 °C. An ultrasonic bath was used for the extraction. This step was repeated for 15 min with 600 mL of the same solvent. The extracts were finally filtered, evaporated and lyophilized, then kept as such in the dark until further use.

### 4.8. HPLC Determination of Betulinic Acid

A betulinic acid stock solution was prepared by dissolving betulinic acid standard in methanol to yield the concentration of 1 mg/mL. Then, a certain amount of stock solution was diluted to solutions with concentrations of 10, 20, 50, 100 and 200 µg/mL with methanol. The equation for the calibration curve of betulinic acid is y = 7.7382x − 90.116. The correlation coefficient of calibration plots was equal to 0.9964. A chromatographic analysis was carried out using an HPLC system (Agilent Technologies 1200 Series, Morges, Switzerland) equipped with a diode array detector (DAD). Chromatographic separation was achieved on an Eclipse XDB C-18 column (4.6 × 150 mm, 5 μm) at 25 °C. The mobile phase consisted of 16% water (A) and 84% acetonitryle (B) in an isocratic mode with an injection volume 20 μL. The flow rate was 1 mL/min and the detection was carried out at 210 nm [59].

Precisely weighed equivalents of samples were dissolved in methanol and sonicated for 30 min at 30 °C in an ultrasonic bath. Then, the samples were centrifuged and aliquots of 20 µL of solution were injected into the HPLC system.

### 4.9. Cell Cultures

The glioblastoma multiforme U87MG (HTB-14; ATCC), human lung adenocarcinoma A549 (CCL-185; ATCC) and human prostate carcinoma DU-145 (HTB-81; ATCC) cell lines used in the experiments were obtained from American Type Culture Collection (ATCC™, Manassas, VA, USA). All cell lines were cultured in a humidified incubator at 37 °C and 5% CO_2_. A549 cells were grown in DMEM medium supplemented with 100 units of Potassium Penicillin and 100 µg of streptomycin sulfate per 1 mL of culture media and 10% (*v*/*v*) heat-inactivated Fetal Bovine Serum (FBS, Gibco, Thermo Fisher, Waltham, MA, USA). The DU-145 cells were subcultured in Dulbecco’s modified eagle medium supplemented with 10% fetal bovine serum, 1% penicillin–streptomycin, 1% nonessential amino acids in tissue culture flasks. U87MG cells were grown in DMEM medium supplemented with 10% (*v*/*v*) heat-inactivated fetal bovine serum (FBS), 100 U/mL of penicillin and 100 μg/mL of streptomycin. All cell culture media and components were purchased from Lonza (Basel, Switzerland).

### 4.10. Cell Viability

An MTT assay was employed to measure the viability of all cell lines treated with transgenic hairy root extract of *S. obtusifolia*. Briefly, cells were seeded at 1 × 10^4^ cells per well in 96-well culture plates and left overnight before treatments for attachment. In the next step, the cells were incubated for 24 h with the tested extract. In the following step, the cells were incubated with 0.5 mg/mL of 3-(4,5-dimethylthiazol-2-yl)-2,5-diphenyl tetrazolium bromide (MTT) at 37 °C for 1.5 h. After this time, MTT was carefully removed and DMSO was added to each well and vortexed at low speed for 5 min to fully dissolve the formazan crystals. Absorbance was measured at 570 nm with a reference at 630 nm using a Bio-Tek Synergy HT Microplate Reader (Bio-Tek Instruments, Winooski, VT, USA), according to our previous studies [17].

### 4.11. Apoptosis/Necrosis Detection by Flow Cytometry

In this experiment, a population of apoptotic and necrotic cells of the U87MG cell line was detected using an Annexin V–fluorescein isothiocyanate (FITC)/propidium iodide (PI) (BD Biosciences, San Jose, CA, USA) detection kit according to the manufacturer’s protocol. Briefly, the tested U87MG cells were plated into 6-well culture dishes (2 × 10^5^ cells/well) for 24 h prior to the addition of hairy root extract of *S. obtusifolia* at the IC_50_ concentration used in this study. Following 24 h incubation with both extracts, the percentage of apoptotic/necrotic cells was determined by the Annexin V–FITC/PI assay, according to our previous studies [60].

### 4.12. Mitochondrial Membrane Potential (MMP)

To measure MMP, the fluorescent probe JC-1 (5’,6,6’-tetrachloro-1,1’,3,3’-tetraethylbenzimidazolylcarbocyanine iodide) was used. Briefly, cells were seeded into black 96-well tissue culture plates with transparent bottoms (Greiner Bio-One, Solingen, Germany) at a density of 1 × 104 cells/well U87MG in 50 µL of culture medium and allowed to adhere overnight; then, they were treated with IC_50_ concentration of hairy root extracts of *S. obtusifolia* for 24 h. Finally, the cells were preincubated with 5 μM JC-1 in the HBSS in a CO_2_ incubator at 37 °C for 30 min. Prior to measurements, the cells were centrifuged (300 g for 10 min at 22 °C), then washed twice with the HBSS. The fluorescence was measured on a Bio-Tek Synergy HT Microplate Reader (Bio-Tek Instruments, Winooski, VT, USA) with the filter pairs of 530 nm/590 nm and 485 nm/538 nm, according to our previous studies. As positive control, we used 10 µM Carbonyl cyanide m-chlorophenylhydrazone (CCCP) [60].

### 4.13. Chromosomal DNA Fragmentation

U87MG cells (5 × 10^5^) were seeded into a 6-well plate and treated with transgenic hairy root extracts of *S. obtusifolia*. Cells were washed once with ice-cold PBS and resuspended in 250 μL of lysis buffer (10 mM Tris–HCl, pH 7.6, 20 mM EDTA, pH 8.0 and 0.5% (*w*/*v*) Triton X-100). After centrifugation at 12,000 rpm for 5 min, the supernatant was extracted once with phenol/chloroform and once with chloroform/isoamyl alcohol. DNA was precipitated with sodium acetate (pH 5.2) at −20 °C overnight. The DNA was then pelleted and subsequently digested with DNase-free RNAase A (Sigma, Sigma Chemical Co., St. Louis, MO, USA) at 37 °C for 20 min. Finally, DNA samples were separated by electrophoresis in 1.5% agarose gels and the results were visualized by trans-illumination with UV light (Vilber Lourmat, Marne-la-Vallée, France), following ethidium bromide staining to determine the extent of apoptotic DNA fragmentation.

### 4.14. DNA Damage Measured by Comet Assay

The cells were treated with a IC_50_ concentration of hairy root extracts for up to 24 h before washing twice with 1 mL of PBS and collecting into 1 mL of PBS; they were analyzed by a neutral version of the comet assay to detect DSBs. Briefly, the cells were suspended in 0.75% LMP agarose and casted onto microscope slides precoated with 0.5% NMP agarose. The cells were then lysed for one hour at 4 °C in a buffer consisting of 2.5 mM NaOH, 100 mM EDTA, 1% Triton X-100 and 10 mM Tris, pH 10. After lysis, the slides were placed in an electrophoresis unit; DNA was left to rest for 20 min in an electrophoresis buffer consisting of 100 mM Tris and 300 mM sodium acetate at a pH adjusted to 9.0 by glacial acetic acid. Electrophoresis was conducted in this electrophoresis buffer at 4 °C for 60 min at an electric field strength of 0.41 V/cm (100 mA). The slides were then washed in water, drained and stained with 2 µg/mL of DAPI and examined with a microscope image analysis system. Further procedure has been described in our previous studies [50,61].

### 4.15. Effect on the Activity of Caspase-3/7

The influence of transgenic hairy root extract of *S. obtusifolia* on caspase-3/7 activity in U87MG cells was observed using Caspase-Glo 3/7 Assay kit (Promega, Madison, WI, USA). According to the manufacturer’s protocol, cells culture media were seeded in 96-well plates overnight, then treated with the IC_50_ of the transgenic hairy roots’ extracts. After 24 h of treatment, 100 µL of caspase reagent was added to each well, mixed and incubated for 1 h at room temperature. The luminescence was measured using a GloMax-Multi Detection System (Promega, Madison, WI, USA). Then, caspase activity was expressed as a percentage of the untreated control within the reading of three replicates.

### 4.16. Topoisomerase I Inhibition Assay with Hairy Root Extract

A topoisomerase I inhibition assay with transgenic hairy root extract was performed as follows: A total of 0.25 μg of supercoiled pUC19 plasmid DNA and 5 units of topoisomerase I were mixed in the reaction buffer (50 mM potassium acetate, 20 mM Tris-acetate, 10 mM magnesium acetate and 100 µg/mL albumin, pH 7.9). The total cleavage reaction volume was 20 μL. Hairy root extract was applied at a IC_50_ concentration. The negative control was performed with water, supercoiled DNA and cleavage buffer; the positive control contained topoisomerase I in the reaction buffer with DNA. The reaction was started by the addition of plant extracts and incubated at 37 °C for 30 min and then terminated by adding 4 µL of stop buffer (3% SDS, 60 mM EDTA, 50% glycerol and 0.25% bromphenol blue). All samples were electrophoresed in 1% agarose gel in TAE at room temperature. The DNA bands were visualized with ethidium bromide under UV light [62].

### 4.17. Statistical Analysis

The results are here expressed as mean values ± SD. To confirm the normality of the data, the Shapiro–Wilk test was used. Significant changes were calculated with the analysis of variance (ANOVA) test with the appropriate *post-hoc* tests as a multiple comparison procedure. The statistical analyses were performed using the STATISTICA 13.3 software for Windows (StatSoft, Krakow, Poland). Differences of *p* < 0.05 were considered statistically significant.

## 5. Conclusions

In this work, we demonstrate, for the first time, an increase in the content of pentacyclic triterpene (betulinic acid) using in vitro culture techniques (10 L sprinkle bioreactor), in combination with metabolic engineering and elicitation with methyl jasmonate in our *S. obtusifolia* transgenic hairy root system.

Based on our previous research studies, we present a potential mechanism of apoptosis induction in glioblastoma cells of the U87MG line by activating the caspase-3/7 cascade, reducing the mitochondrial potential, inducing DNA fragmentation and increasing the level of DNA damage.

## Data Availability

Not applicable.

## Supplementary Materials

The following are available online, Figure S1: Representative HPLC chromatograms of (a) betulinic acid standard and (b) transgenic hairy roots of *S. obtusifolia* after MeJA treatment extract detected at 210 nm.

## Sample Availability

Samples of the compounds are available from the corresponding author.
