# Supplementary material for: Methyl Jasmonate Effect on Betulinic Acid Content and Biological Properties of Extract from Senna obtusifolia Transgenic Hairy Roots
Source: Molecules. 2021 Oct 14;26(20):6208. doi: 10.3390/molecules26206208 (PMC8540613; doi:10.3390/molecules26206208)
Supplement: Supplementary file 1 [file molecules-26-06208-s001.zip › molecules-1399267-supplementary.pdf]

molecules

MDPI

Supplementary Materials

Methyl Jasmonate Effect on Betulinic Acid Content and Biological Properties of Extract from *Senna obtusifolia* Transgenic Hairy Roots

Tomasz Kowalczyk [1,*], Przemysław Sitarek [2], Anna Merecz-Sadowska [3], Monika Szyposzyńska [4], Aleksandra Spławska [4], Leslaw Gorniak [5], Michał Bijak [5] and Tomasz Śliwiński [6]

[1] Department of Molecular Biotechnology and Genetics, University of Lodz, Banacha 12/16, 90-237 Lodz, Poland
[2] Department of Biology and Pharmaceutical Botany, Medical University of Lodz, Muszynskiego 1, 90-151 Lodz, Poland; przemyslaw.sitarek@umed.lodz.pl
[3] Department of Computer Science in Economics, University of Lodz, 90-214 Lodz, Poland; anna.merecz-sadowska@uni.lodz.pl
[4] CBRN Reconnaissance and Decontamination Department, Military Institute of Chemistry and Radiometry, Antoniego Chrusciela "Montera" 105, 00-910 Warsaw, Poland; m.szyposzynska@wichir.waw.pl (M.S.); a.splawska@wichir.waw.pl (A.S.)
[5] Biohazard Prevention Centre, Faculty of Biology and Environmental Protection, University of Lodz, Pomorska 141/143, 90-236 Lodz, Poland; leslaw.gorniak@biol.uni.lodz.pl (L.G.); michal.bijak@biol.uni.lodz.pl (M.B.)
[6] Department of Medical Biochemistry, Medical University of Lodz, Mazowiecka 6/8, 92-215 Lodz, Poland; tomasz.sliwinski@biol.uni.lodz.pl
* Correspondence: tomasz.kowalczyk@biol.uni.lodz.pl

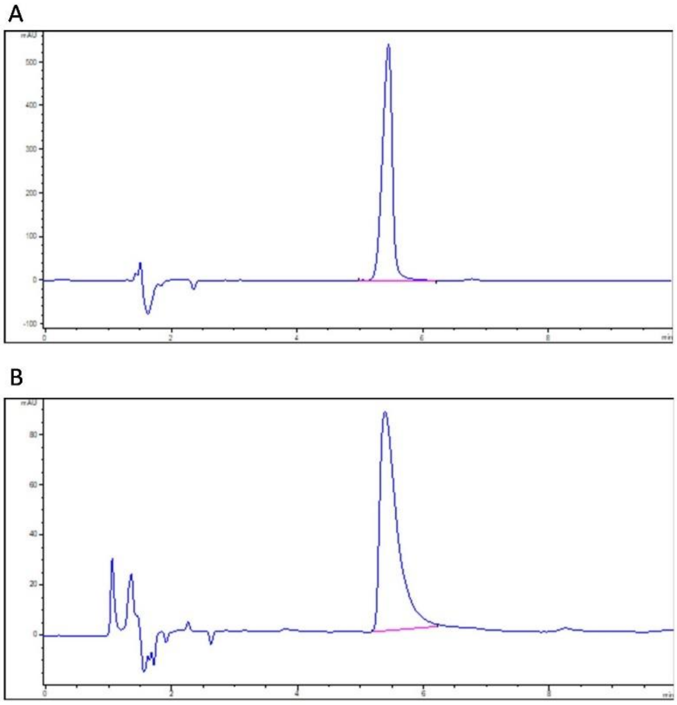

Figure S1. Represantive HPLC chromatograms of (**a**) betulinic acid standard and (**b**) transgenic hairy roots of *S. obtusifolia* after MeJA treatment extract detected at 210 nm.
